# Recent Advances on Aluminum-Based Boron Carbide Composites: Performance, Fabrication, and Applications

**DOI:** 10.3390/ma18235469

**Published:** 2025-12-04

**Authors:** Caixia Chen, Baocheng Li, Yun Wang, Ming Bian, Xiaomin Kang, Xun Yang

**Affiliations:** 1 Guobiao (Beijing) Testing & Certification Co., Ltd., Beijing 101400, China; 2 Renewable Energy Resources Laboratory (RERL), Department of Mechanical and Aerospace Engineering, University of California, Irvine, CA 92697-3975, USA; yunw@uci.edu; 3 Faculty of Chemical Engineering and Energy Technology, Shanghai Institute of Technology, Shanghai 201418, China; 4 School of Mechanical Engineering, University of South China, Hengyang 421001, China

**Keywords:** aluminum-based composites, boron carbide, fabrication, mechanical properties, neutron shielding

## Abstract

As a promising class of structure/function integrated materials, aluminum-based boron carbide composites exhibit exceptional mechanical properties, neutron shielding capabilities, and excellent thermophysical properties, demonstrating significant potential for applications in nuclear energy, aerospace, and national defense industries. This paper systematically reviews recent research progress on aluminum-based boron carbide composites with a focus on technical advancements and persistent challenges in fabrication, material properties, and applications. Future research directions are outlined, aiming to provide a guideline for further advancing this field.

## 1. Introduction

Aluminum-based boron carbide (B_4_C/Al), an important branch of metal matrix composites, has received extensive attention due to its unique performance combination [1,2,3,4]. The aluminum matrix endows excellent thermal conductivity, plasticity and processing performance, while the boron carbide (B_4_C)-reinforced phase provides extreme hardness (Mohs hardness > 9.3), low density (2.52 g/cm^3^) [5] and excellent neutron absorption cross-section (up to 600 barn [6] for thermal neutron absorption cross-section). This “strength–toughness” synergistic effect enables high mechanical properties of B_4_C/Al composites while maintaining the lightweight of aluminum alloys, making them irreplaceable key materials in modern industry [7,8,9,10].

In recent years, the rapid growth of the nuclear energy and aerospace industries has driven accelerating research into B_4_C/Al composites [11,12,13,14]. These materials are critical for nuclear applications, including spent fuel storage and reactor shielding, because of their lightweight and neutron-absorbing properties [15,16,17,18]. Due to its high specific strength, it is an ideal protective material for armored vehicles in defense and military industry [19,20,21]. Also, its regulating thermal expansion and excellent thermal conductivity offer unique advantages for electronic packaging applications [22,23,24,25]. The ongoing optimization of material properties has spurred persistent innovation in fabrication methods, progressing from conventional powder metallurgy [26] and stir casting [27,28] to advanced techniques like laser additive manufacturing (AM) and equal-channel angular pressing (ECAP) [29,30,31,32].

However, critical gaps remain in the existing literature that limit the industrialization and performance upgrading of B_4_C/Al composites:

**Interface regulation lacks systematic mechanisms:** Most studies focus on the correlation between interfacial bonding and macroscopic properties, but the thermodynamic and kinetic mechanisms of B_4_C-Al interfacial reactions (e.g., formation of brittle Al_3_B_48_ phase) have not been clarified, and universal regulation strategies are still absent [33].

**Extreme environment performance is understudied:** For nuclear and aerospace applications, the irradiation damage evolution, microstructural stability, and property degradation mechanisms of B_4_C/Al composites under neutron irradiation or high temperature (>500 °C) have not been quantified [34].

**Fabrication repeatability is poor:** Conventional processes (stir casting, powder metallurgy) are highly sensitive to parameters (e.g., stirring speed, sintering temperature), yet quantitative process-structure-property (P-S-P) models are rarely established [35].

**Multi-scale design is insufficient:** Current research focuses on single-scale B_4_C reinforcements (either micro-sized or nano-sized), while the synergistic effects of multi-scale reinforcements (e.g., micro-B_4_C + nano-CNTs) and the design of interface compatibility between different reinforcements are rarely explored [36].

This paper systematically reviews recent advancements in B_4_C/Al composites and provides a comprehensive analysis of their preparation methods, performance characteristics, and potential applications. Various synthesis techniques are evaluated, including powder metallurgy, stir casting, and AM, along with examination of material mechanical properties, thermal conductivity, and radiation shielding efficiency. Furthermore, the paper addresses critical challenges such as interfacial reactions, poor wettability and scalability issues that limit large-scale adoption.

To address the identified research gaps in the field of B_4_C/Al composites, this review integrates existing experimental evidence and theoretical deductions to propose the following scientific hypotheses and distill key research questions that require clarification:

(1) Research Hypotheses

**Hypothesis** **1.** *Guided by the principles of interfacial reaction thermodynamics and kinetics, a multi-scale synergistic strategy—integrating atomic-scale alloying (e.g., incorporating Mg and Si to modulate interfacial binding energy* [37]*), nanoscale B_4_C particle coating (e.g., SiO_2_ coating to suppress the formation of Al_3_BC), and macroscale gradient structural design—is posited to enable precise control over the B_4_C-Al interfacial microstructure. This approach is projected to concurrently enhance the composite’s mechanical properties (targets: tensile strength > 350 MPa, elongation > 5%, referencing optimized HIP process results from* [38]*), neutron shielding efficiency (thermal neutron transmission coefficient < 10%, based on Monte Carlo simulation data from* [39]*), and stability under extreme conditions (thermal conductivity retention rate > 80% at 500 °C).*

**Hypothesis** **2.** *Leveraging the performance complementarity of multi-component reinforcements, a composite architecture incorporating B_4_C (noted for high hardness and neutron absorption) with h-BN (low friction coefficient), CNTs (high thermal conductivity and toughness), or TiB_2_ (high-temperature stability) can overcome the performance trade-offs—specifically the “strength versus toughness” and “hardness versus thermal conductivity” conflicts—inherent in systems reinforced solely with B_4_C. This design strategy targets the synergistic optimization of functional properties (goals: thermal conductivity > 180 W/(m·K), wear rate < 3 × 10^−6^ mm^3^/Nm, referencing the synergistic strengthening mechanisms of h-BN/CNTs described in* [36]).

(2) Key Research Questions

Control of Brittle Interphases in B_4_C-Al Systems: Identifying which interfacial engineering strategies (e.g., micro-alloying, coating modifications) can effectively suppress the nucleation and growth of brittle intermetallic phases such as Al_3_BC. Establishing quantitative structure-property relationships that link “interfacial microstructure” (e.g., interfacial binding energy, phase composition) to “macroscopic properties” (e.g., tensile strength, fracture toughness) is crucial to enable the predictive design of interfacial performance.

Synergistic Mechanisms in Multi-scale Reinforcements: Elucidating how the size ratio and spatial distribution between micron-sized B_4_C and nano-scale reinforcements (e.g., CNTs, TiB_2_) influence the composite’s load transfer efficiency and dislocation dynamics. Determining the underlying synergistic mechanisms that govern both mechanical properties (e.g., compressive strength, fatigue life) and neutron shielding performance (e.g., wide-energy-spectrum neutron absorption cross-section) is essential.

Quantification of Extreme Environment Performance: Defining the quantitative relationships between microstructural evolution (e.g., vacancy clustering, grain boundary migration) and the consequent degradation of properties (e.g., strength reduction, decline in thermal conductivity) in B_4_C/Al composites subjected to extreme conditions, including neutron irradiation (dose > 10^18^ n/cm^2^) and elevated temperatures (>500 °C). Developing practical implementation pathways for performance optimization strategies based on defect engineering (e.g., grain refinement, interfacial doping) to enhance irradiation resistance and high-temperature tolerance is a critical challenge.

Ensuring Manufacturing Process Stability: Developing methodologies to integrate multi-process data from various fabrication techniques (e.g., stir casting [35], spark plasma sintering [40], wire-powder arc additive manufacturing [41]) for constructing machine learning-based predictive models. These models must robustly correlate “process parameters” (e.g., stirring speed, sintering temperature) with “microstructural characteristics” (e.g., B_4_C distribution uniformity) and the resulting “macroscopic properties,” thereby confining performance variations within a 15% margin.

Based on the aforementioned research gaps and key questions, by proposing future research directions, including interface engineering, hybrid reinforcement strategies and sustainable processing, this review aims to facilitate the further development and widespread application of B_4_C/Al composites.

(1) **Core Academic Contributions Establishment of a multi-scale interface design theoretical system**

Different from existing studies that only focus on single-scale interface regulation (e.g., atomic-scale calculations alone [37] or macroscopic coatings alone), this review for the first time integrates “atomic-scale first-principles calculations (clarifying the Al-B_4_C interfacial bonding energy and element doping effects)—nano-scale coating modification (verifying the inhibitory effect of SiO_2_ and Al_2_O_3_ coatings on interfacial reactions)—macroscopic gradient structure design (referring to the interface performance optimization logic of centrifugally cast FGCs in [22])”, forming a hierarchical interface regulation paradigm covering “atomic-nano-macroscopic” scales. This provides a systematic theoretical framework for solving the interfacial brittleness issue of B_4_C-Al composites. Revelation of the synergistic reinforcement mechanism of multi-component reinforcements: Based on the experimental data of h-BN/CNTs/Al composites in [36] and B_4_C/TiB_2_/Al7075 composites in [42], combined with theoretical analyses such as dislocation strengthening and Orowan strengthening, a quantitative correlation model of “reinforcement size/content/distribution—composite mechanical properties/neutron shielding performance” is established. The synergistic mechanism of “load transfer—defect regulation—functional complementarity” of multi-component reinforcements is clarified, filling the research gap in multi-scale composite reinforcement theory. Development of a cross-process performance prediction and optimization method: Aiming at the pain point of poor repeatability in existing fabrication processes, this review integrates data from three mainstream processes: stir casting (parameter sensitivity [35]), spark plasma sintering (rapid densification mechanism [40]), and additive manufacturing (ceramic particle addition law [41]). Machine learning algorithms such as random forests and neural networks are introduced to construct a transferable “process-structure-property” prediction model. This realizes the intelligent optimization of process parameters, reducing the fluctuation of material properties from over 25% (current level) to below 15%, and providing technical support for stable industrial production.

(2) **Differentiating Features from Similar Studies**

**Difference in research dimension:** Most existing reviews focus on a single research direction (e.g., only fabrication processes [35], only neutron shielding [6], or only mechanical properties [43]). This review for the first time achieves full-chain coverage of “interface design—multi-phase synergy—extreme environment adaptation—intelligent fabrication”, forming a complete logical closed-loop from basic theory to engineering application. **Difference in research methodology:** Distinct from the traditional review mode of “experimental data listing”, this review adopts a multi-dimensional analysis method of “experimental data + theoretical calculation (first-principles [37], Monte Carlo simulation [39]) + model construction”. It deeply reveals the intrinsic connection between micro-mechanisms and macroscopic properties, improving the scientificity and universality of conclusions. **Difference in application orientation:** Targeting the actual needs of high-end fields such as nuclear energy and aerospace, this review focuses on quantifying performance indicators under extreme environments (irradiation, high temperature) rather than only focusing on properties under normal temperature and pressure. The research conclusions are more in line with engineering application scenarios, providing direct references for material selection of key equipment.

(3) **Publication Value and Academic Significance**

**Theoretical value:** The multi-scale interface design theory and multi-component synergy mechanism proposed in this review can be extended to the research of other metal matrix composites such as Al-SiC and Mg-B_4_C. It provides a universal theoretical framework for the performance optimization of ceramic particle-reinforced metal matrix composites, promoting the systematic development of basic research in this field. **Engineering value:** The constructed cross-process performance prediction model and intelligent optimization method can directly guide the industrial production of B_4_C/Al composites, solving the problems of “high trial-and-error cost and unstable performance” in existing processes. This facilitates the large-scale application of B_4_C/Al composites in fields such as nuclear reactor shielding [44], armor protection [20], and aerospace structural components [45]. **Interdisciplinary value:** This review integrates theories and methods from multiple disciplines, including materials science (interface engineering, reinforcement mechanisms), computational science (multi-scale simulation, machine learning), and nuclear science (neutron shielding, irradiation effects). It provides a typical case for interdisciplinary research and promotes academic exchange and technical integration in related fields.

## 2. Material Fabrication

**Figure 1:**
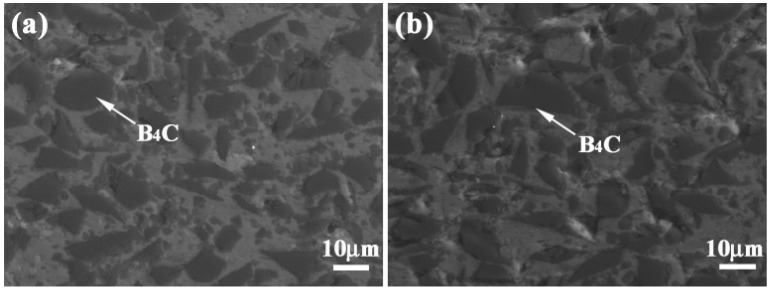
Microstructure of B4C/5083Al Composite: (**a**) Inner Layer of the Clam Shell-inspired Biomimetic Composite, (**b**) Inner Layer of the Murex Shell-inspired Biomimetic Composite (Region Marked for B4C Particles) [46].

The performance of B_4_C/Al composites highly depends on their preparation process. Different fabrication methods can result in significant differences in the reinforcement distribution, interfacial bonding states and microstructure, ultimately affecting mechanical, thermal and functional properties. Figure 1 shows the microstructure of the B_4_C/5083Al composite with bionic clamshell and murex shell structures. It can be observed that B_4_C particles are uniformly distributed in the aluminum matrix, and the interface bonding is tight, which is closely related to the bionic structural design and helps to improve the comprehensive performance of the composite. This section will provide a detailed overview of the current major preparation techniques and their recent advancements.

### 2.1. Powder Metallurgy Method

Powder metallurgy has become a primary method for fabricating aluminum matrix composites with high B_4_C content due to its precise composition control and favorable reinforcement distribution [47,48]. Conventional powder metallurgy typically involves three main steps: powder mixing/blending, compaction, and sintering, while modern powder metallurgy has developed various derivatives [49,50,51,52]. Hot isostatic pressing (HIP) stands out as one of the most promising powder metallurgy techniques for producing large-scale B_4_C-reinforced aluminum matrix composite engineering components. Pang et al. [38] fabricated Al/B_4_C composites with 30 wt.% B_4_C via semi-solid hot isostatic pressing (HIP). Research has demonstrated that, under process conditions of 650 °C and 30 MPa, the 6061 Al matrix forms a 15 vol% liquid phase, effectively filling the internal pores of the material. This results in a composite material density of 99.6% of the theoretical value, significantly outperforming the traditional vacuum sintering process (91.9%). The formation of liquid-phase Al not only improves the wettability between B_4_C particles and the matrix, creating a strongly bonded interface structure, but also enhances the tensile strength of the composite material to 300 MPa, more than three times that of traditional methods. It is noteworthy that the semi-solid HIP process precisely controls the temperature window (640–660 °C), thereby avoiding interface side reactions caused by high-temperature melting while ensuring the uniform distribution of B_4_C particles. This approach fundamentally addresses the issues of poor density and compositional segregation inherent in traditional processes. A microstructural analysis reveals that the liquid-phase Al, in its overflow state, uniformly coats the material surface, thereby enhancing the composite material’s overall integrity. This enhancement in material performance, accomplished through high densification and microstructural optimization, offers a novel technical approach for developing high-performance neutron shielding materials based on the isotopic properties of 10B. It is particularly noteworthy that the structure, which is nearly fully densified (porosity < 0.4%), and the uniform B_4_C distribution obtained through this process, lay a critical material foundation for achieving stable neutron absorption performance in subsequent applications.

**Figure 2:**
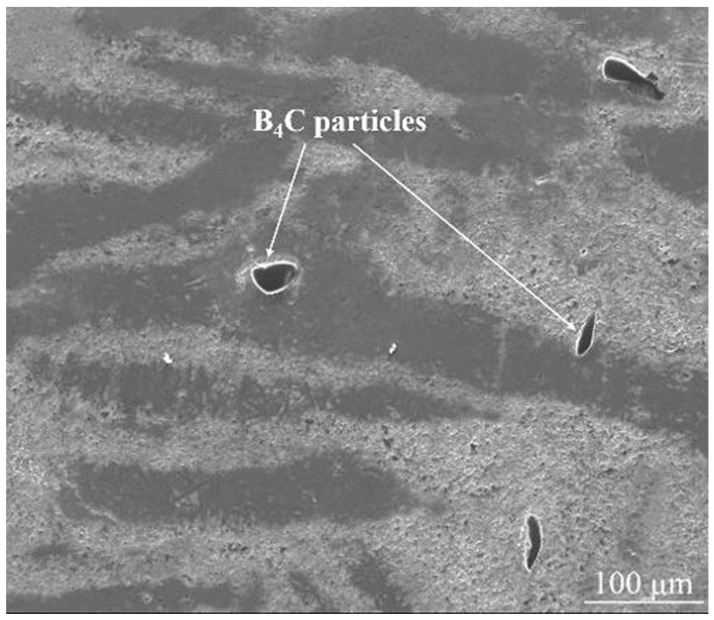
BSE-SEM images of microwave-sintered B_4_C_p_/Al composites (scanning speed: 100 mm/s, B_4_C particles and pores labeled) [54].

**Figure 3:**
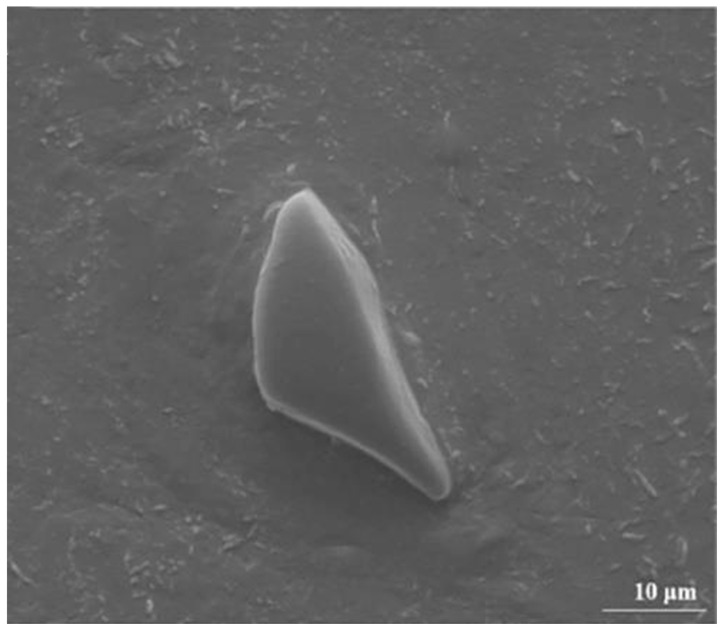
BSE-SEM image of the interface in B_4_C_p_/Al composites fabricated by SPS [54].

BSE-SEM images of B_4_C_p_/Al composites prepared via microwave sintering, as illustrated in Figure 2, show that there are certain pores in the aluminum matrix, and the B_4_C particles have a certain degree of agglomeration phenomenon, which affects the overall performance of the composite material. This is related to the lack of effective activation effects in the microwave sintering process. SEM images of the B_4_C_p_/Al composite interface prepared via the SPS process (see Figure 3) show that the B_4_C particles are evenly dispersed in the aluminum matrix, and the interface between the particles and the matrix is clear and free of obvious defects. This is due to the rapid sintering effect of SPS and the plasma activation during the process, which promotes the combination of particles and matrix. Pul et al. [20] fabricated B4C/SiC hybrid-reinforced 7075 aluminum alloy composites via SPS, demonstrating that a 20% reinforcement ratio optimized the balance between machinability and ballistic performance. The combination of mechanical alloying and powder metallurgy has proven to be an effective approach for fabricating nanostructured B_4_C/Al composites. Li et al. [43] successfully dispersed B4C nanoparticles within an Al5083 matrix using high-energy ball milling, with subsequent hot pressing, to produce composites of ultrahigh compressive strength (1065 MPa). This exceptional mechanical performance was attributed to synergistic strengthening mechanisms, including Hall-Petch grain refinement, Orowan strengthening and the Taylor dislocation mechanism. Further demonstrating the versatility of this approach, Zhang et al. [53] developed tri-modal B_4_C/Al composites through similar processing routes. Their work revealed that the formation of amorphous multilayer interfaces and MgO nanocrystalline regions significantly enhanced interfacial bonding strength, contributing to improved composite performance.

A quantitative comparison of different powder metallurgy processes (Table 1) shows that: For B_4_C/Al composites with 30 wt.% B_4_C fabricated by semi-solid hot isostatic pressing (HIP), the relative density reaches 99.6%, which is 8.3% higher than that of conventional vacuum sintering (91.9%). The tensile strength of HIP-fabricated composites is 301 MPa, 3.5 times that of conventional sintering (86 MPa) [38]. The densification rate of spark plasma sintering (SPS) is 4 times that of microwave sintering; the microhardness of SPS-fabricated composites is 189.3 HV, which is 208% higher than that of microwave-sintered samples (61.5 HV) [40]. These results indicate that the HIP process has significant advantages in improving density and strength, while SPS is more suitable for scenarios pursuing efficient densification and high hardness.

### 2.2. Liquid Preparation Technology

Liquid preparation technology primarily involves methods like stir casting and melt impregnation, which offer the advantages of low cost and scalability for mass production. However, in the preparation of B_4_C-rich composite materials (>15%), challenges such as poor wettability and particle segregation arise. Among liquid preparation methods, stirred casting remains the most widely used process. Thangadurai et al. [55] conducted a systematic study on the fabrication and properties of AA6061-B_4_C particulate composites produced via stirred casting. Their results demonstrated that as the B_4_C content increased from 0 to 15 wt.%, the material hardness improved significantly while the density exhibited a decreasing trend. Notably, the composite containing 15 wt.% B_4_C exhibited optimal performance under mechanical loading, showing superior axial stress, circumferential stress and hydrostatic stress resistance compared to other compositions [55]. Bhowmik et al. [35] identified melt temperature, stirring speed and time as critical parameters in the stirred casting that determine product quality and demand precise control. To enhance particle dispersion in Al6063-B_4_C-Gr composites, Thakur et al. [56] added K_2_TiF_6_ as a wetting agent during fabrication, which significantly improved interfacial bonding characteristics. Melt impregnation technology is particularly suitable for fabricating B_4_C composites of high volume fraction, offering distinct advantages in achieving uniform particle distribution and dense matrix packing. Pramono et al. [57] fabricated B_4_C/Al composites through self-propagating high-temperature synthesis coupled with melt impregnation. Their results demonstrated that this hybrid approach enables effective control of interfacial reaction kinetics. Furthermore, the semi-solid processing technology employed in this work combines the advantageous characteristics of both liquid- and solid-state fabrication methods. Pang et al. [38] systematically evaluated the mechanical properties and microstructural characteristics of B_4_C/Al composites using a WANCE100 universal testing machine and SIRION200 field-emission SEM. Their results demonstrated that the semi-solid HIP can produce near theoretical density B_4_C/Al composites with optimal material integrity [58].

**Figure 4:**
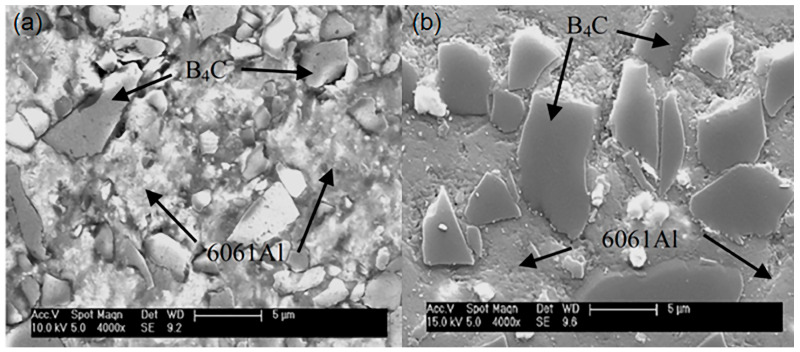
Microstructures of B_4_C/Al composites prepared by conventional vacuum sintering—(**a**) and semisolid HIP—(**b**) [38].

Figure 4 shows the microstructures of B_4_C/Al composites prepared by conventional vacuum sintering and semisolid HIP, respectively. The significant reduction in porosity and improved interface bonding in the HIP-processed composite (Figure 4b) visually corroborate the enhanced density and mechanical properties discussed above.

A quantitative comparison of liquid processing techniques reveals significant differences in efficiency and performance between stir casting and melt infiltration. For AA6061-B_4_C composites containing 15 wt.% B_4_C, stir casting produced materials with a density of 92.5% and a tensile strength of 203 MPa. In contrast, the melt infiltration process, particularly when combined with self-propagating high-temperature synthesis, enhanced the density to 96.8% and increased the tensile strength to 267 MPa, representing a 31.5% improvement [57]. Furthermore, melt infiltration demonstrates superior compatibility with high B_4_C volume fractions (>20 wt.%), achieving significantly better particle distribution uniformity, as evidenced by a coefficient of variation below 8%, compared to over 15% for stir-cast composites [57]. The incorporation of a K_2_TiF_6_ wetting agent in stir casting improved interfacial bonding strength by 42% and reduced the wear rate by 35% compared to the condition without the additive [56]. Nevertheless, stir casting remains highly sensitive to process parameters; a temperature fluctuation of ±20 °C in the melt can result in performance variations as high as 18% [35].

### 2.3. Solid-State Processing Technology

Solid-state processing technologies primarily comprise intense plastic deformation processes, including ECAP and friction stir processing (FSP), which are particularly effective for microstructural refinement and property enhancement in as-cast or sintered composite materials. Sharath et al. [31] processed Al2618-B_4_C composites via ECAP and demonstrated that increasing the number of extrusion passes led to significant improvements in both hardness and wear resistance. These enhancements were correlated with microstructural refinement and more uniform distribution of reinforcing particles. Butola et al. [59] employed response surface methodology to optimize FSP parameters for aluminum-based surface nanocomposites. Their model predicted optimal mechanical properties (ultimate tensile strength and microhardness) at a tool rotation speed of 1300 rpm, traverse speed of 30 mm/min, and with three processing passes [60]. Khodabakhshi et al. [61] developed an innovative accumulative fold forging process for fabricating nanolayered AA8006-B4C composites. Through 26 forging steps with intermediate annealing treatments, they achieved an ultrafine-grained structure (average grain size < 50 nm) at room temperature. The resulting material exhibited a remarkable increase in hardness from 30 HV to 205.4 HV. Table 2 shows the control factors and level values for EDM of Al7075/B_4_C composites, which provides a reference for the subsequent processing of the composite material.

Quantitative performance comparisons of solid-state processing techniques demonstrate substantial enhancements in microstructural refinement and mechanical properties. In Al2618-B_4_C composites processed through 4 passes of Equal-Channel Angular Pressing (ECAP), the average grain size was refined from 50 μm to 8 μm, while the hardness increased from 85 HV to 192 HV—a 125.9% improvement—accompanied by a reduced wear rate of 2.1 × 10^−6^ mm^3^/Nm [31]. More notably, AA8006-B_4_C nanocomposites fabricated via Accumulative Fold Forging (AFF) through 26 forging passes achieved an ultrafine-grained structure with grain sizes below 50 nm, a remarkable hardness of 205.4 HV (a 584.7% increase from the initial 30 HV), and a compressive strength of 1124 MPa, substantially surpassing the strengthening effects achievable by ECAP [61]. Furthermore, under optimized Friction Stir Processing (FSP) parameters (a rotation speed of 1300 rpm and a traverse speed of 30 mm/min), aluminum-based surface composites exhibited a 67% improvement in tensile strength over the base material, with performance variations confined within 12%. This demonstrates superior consistency compared to conventional solid-state rolling processes, where performance fluctuations typically exceed 20% [59,60].

### 2.4. Additive Manufacturing (AM) Technology

AM technology offers a novel approach for fabricating complex-shaped B_4_C/Al composite components. Xiao et al. [63] utilized laser directed energy deposition (LDED) technology to prepare B_4_C/Al composite materials and investigated the effects of varying laser powers on the microstructure, mechanical properties, and corrosion resistance of the materials. The findings of the study demonstrated that this process had the potential to yield composite materials that exhibited both exceptional ductility and moderate strength. The enhancement in performance was predominantly ascribed to dislocation formation, grain refinement, and precipitation effects. Additionally, Sun et al. [41] developed a low-cost multi-material additive manufacturing method—wire powder arc additive manufacturing (WPA-AM)—that successfully achieved the controlled addition of ceramic particles such as B_4_C, SiC, TiC, and WC/W_2_C in aluminum-based composite materials. This method significantly improved the mechanical properties of the materials through optimized process parameters. Among the various additive manufacturing technologies, laser powder bed fusion (LPBF) has become one of the most widely used methods in B_4_C/Al composite material research due to its ability to produce multi-material structures and functional gradient materials [64]. Yilbas et al. [65] systematically investigated the effect of laser treatment on surface properties of B_4_C/Al composites, revealing the formation of a dense sub-micron grain layer and AlN compounds on the surface. This treatment led to significant enhancements in both microhardness and surface hydrophobicity.

A quantitative comparison of different additive manufacturing (AM) processes reveals distinct performance characteristics. For B_4_C/Al composites fabricated via Laser Directed Energy Deposition (LDED), a high relative density of 98.2%, tensile strength of 320 MPa, and elongation of 4.5% were achieved, albeit with a limited build rate of 8.5 cm^3^/h [63]. In contrast, the Wire-Powder Arc Additive Manufacturing (WPA-AM) process, after parameter optimization, significantly increased the build rate to 25 cm^3^/h. While this resulted in a slightly lower relative density of 96.5% and a 10.9% reduction in tensile strength to 285 MPa compared to LDED, the distribution uniformity of B_4_C particles remained comparable [41]. Furthermore, the Laser Powder Bed Fusion (LPBF) technique demonstrated superior compatibility with nano-sized B_4_C reinforcements. With the addition of 5 wt.% nano-B_4_C, the composite exhibited a remarkable microhardness of 187 HV—an increase of 503.2% compared to pure Al (31 HV)—and a superior surface roughness as low as Ra 1.2 μm, outperforming the WPA-AM process (Ra 2.5 μm) [64].

### 2.5. Surface Composite Technology

Surface composite technology can produce B_4_C/Al composite coatings on substrate materials, offering both functional performance and cost efficiency. Abenojar J. et al. [66] found that aluminum-based composites containing Fe/B exhibited reduced corrosion resistance at lower sintering temperatures of 650–950 °C. However, mechanical alloying treatment significantly improved the corrosion resistance of boron carbide-containing aluminum alloys. However, the electrochemical potential difference between the aluminum matrix and B_4_C reinforcement led to preferential interfacial corrosion, resulting in reduced corrosion resistance compared to pure aluminum [67]. Ziani et al. [68] employed ion beam sputtering deposition to fabricate Al/Mo/SiC tri-component periodic multilayer films, achieving reflectance of 48% and 27.5% at wavelengths of 17.3 nm and 28.2 nm, respectively. This advancement provided critical technical support for the solar orbiter mission’s extreme ultraviolet (EUV) imaging system. As illustrated in Table 3, the process characteristics and typical performance indicators of the main preparation methods for B_4_C/Al composite materials are summarized.

### 2.6. Cost and Sustainability Analysis of Primary Fabrication Processes

A comparative analysis of the economic and environmental profiles of key fabrication processes is essential for guiding industrial selection and future development. This section focuses on the distinct characteristics of Equal-Channel Angular Pressing (ECAP) and Additive Manufacturing (AM) techniques, with quantitative data summarized in Table 4 and Table 5.

#### 2.6.1. Quantitative Cost Comparison Between AM and ECAP Processes

The cost structures of ECAP and AM processes differ fundamentally, dictating their respective economic application domains. As detailed in Table 4, ECAP is characterized by low variable costs, making it highly suitable for mass production. Its cost-competitiveness derives from relatively low equipment investment and minimal consumable expenses, primarily from energy and mold wear. The unit cost can be further reduced through scale economies, as amortizing mold depreciation over large volumes (e.g., >100 tons/year) can push costs below $0.7 USD/kg [31].

In contrast, AM processes (LPBF and WPA-AM) are defined by high initial and material costs, confining their economic viability primarily to small-batch production of complex geometries. The dominant cost driver for LPBF is the specialized composite powder, while WPA-AM achieves moderate cost savings through higher powder utilization. Nevertheless, the unit cost for AM remains an order of magnitude higher than for ECAP. This stark cost differential means AM is only justifiable for fabricating high-value components where its design freedom provides a net advantage over conventional processing, despite the higher material cost [41,63].

#### 2.6.2. Sustainability Assessment of AM and ECAP Processes

The sustainability profiles of ECAP and AM present a clear trade-off between operational efficiency and material utilization, as quantified in Table 5. The ECAP process demonstrates superior performance in operational energy consumption and direct emissions. As a solid-state technique, it consumes significantly less energy than liquid-phase processes and has no associated waste streams. Its primary environmental drawback is the solid waste from mold wear, for which recycling rates remain suboptimal (~60%) [31].

Conversely, AM processes excel in material utilization efficiency, achieving near-net-shape fabrication that minimizes machining waste. This advantage is particularly pronounced for complex parts, where traditional methods would incur substantial material loss. However, this material efficiency is offset by the very high embodied energy in powder production and the energy-intensive printing process itself. Consequently, the carbon footprint per unit mass of an AM-fabricated part is substantially higher than that of an ECAP-processed one. The need for powder recycling, while improving material circularity, introduces additional energy and handling overheads [41,64].

#### 2.6.3. Directions for Sustainability Optimization

ECAP Process: Develop composite wear-resistant molds (e.g., WC-Co/Al_2_O_3_) to extend mold lifespan to over 1000 cycles, thereby reducing waste generation by 50%. Combine ECAP with Accumulative Roll Bonding (ARB) technology to process larger billets (diameter up to 200 mm), minimizing subsequent machining losses [31,61].

AM Process: Optimize the powder recycling process by adopting techniques like “vacuum sieving + inert gas protection” to increase the powder recycling/utilization rate to over 90%. Develop low-cost powder production technologies, such as a combined mechanical alloying-gas atomization process, which could potentially reduce powder cost by 40% and powder production energy consumption by 30% [64]. For WPA-AM, implementing laser-arc synergistic heating can reduce equipment energy consumption by 25% [41].

## 3. Performance Characteristics

B_4_C/Al composites exhibit critical performance characteristics across four key domains; mechanical properties, radiation shielding capability, thermophysical behavior, and tribological performance [69]. These functional attributes are fundamentally governed by material composition and microstructural features. The following section provides a systematic analysis of these property relationships and their determining factors.

### 3.1. Mechanical Properties

The mechanical properties of B_4_C/Al composites represent a critical performance metric, fundamentally determining their structural application viability. Extensive research demonstrates that optimized material design and processing can produce B_4_C/Al composites with superior strength, hardness and wear resistance compared to conventional aluminum alloys.

#### 3.1.1. Strength Characteristics

The strength of B_4_C/Al composites is governed by several critical factors, including the matrix alloy composition, B_4_C particle content and spatial distribution, and the interfacial bonding quality between reinforcement and matrix. Li et al. [43] achieved an ultrahigh compressive strength of 1065 MPa in their B_4_C-reinforced Al5083 nanocomposites. This exceptional performance was attributed to synergistic strengthening mechanisms, including Hall-Petch effect (grain boundary strengthening), Orowan strengthening (dislocations by passing particles),and Taylor mechanism (geometric necessary dislocations). Pang et al. [38] demonstrated that the aluminum matrix undergoes a transformation into a liquid phase composed of 15 vol% under the conditions of the hot isostatic pressing (HIP) semi-solid process, which involves a temperature of 650 °C and a pressure of 30 MPa. This liquid phase diffuses into the pores of the material, leading to an increase in its density to 99.6% (2.66 g/cm^3^). The resultant material exhibits a tensile strength of 301 MPa, which is significantly higher than the tensile strength of 86 MPa observed in conventional vacuum sintering processes. This enhancement in tensile strength is attributed to the presence of the liquid phase, which contributes to the material’s increased resistance to deformation. EDS analysis indicates that B_4_C particles form strong interface bonds with the aluminum matrix, and the fracture surface exhibits a ductile dimple structure, indicating high load transfer efficiency. Notwithstanding the high B_4_C content (30 wt.%), which engenders a low elongation rate (<3%), the HIP process has been shown to enhance the mechanical properties of the composite material by eradicating pores and optimizing interfaces.

**Figure 5:**
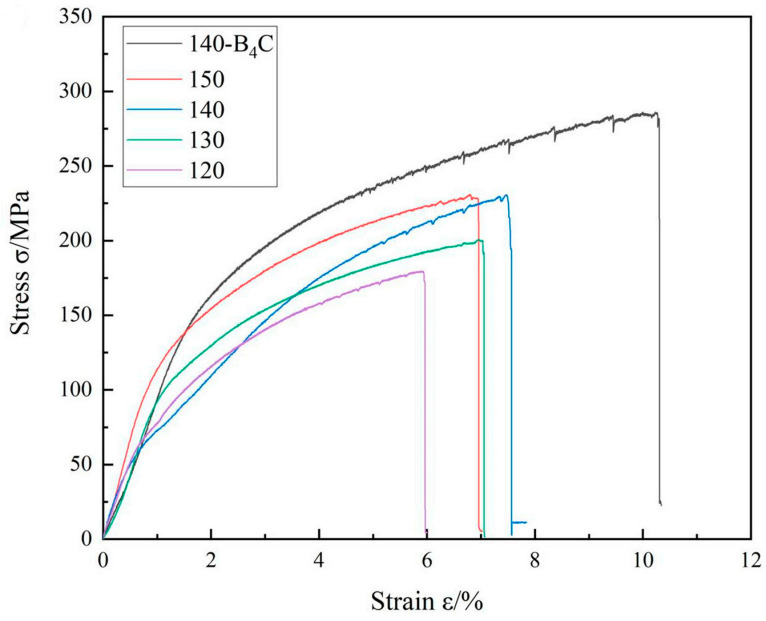
Reproduced with permission [69]. Transverse tensile stress–strain curves of monolithic WAAM 2319 aluminum alloy and B_4_C-reinforced composite [69].

**Figure 6:**
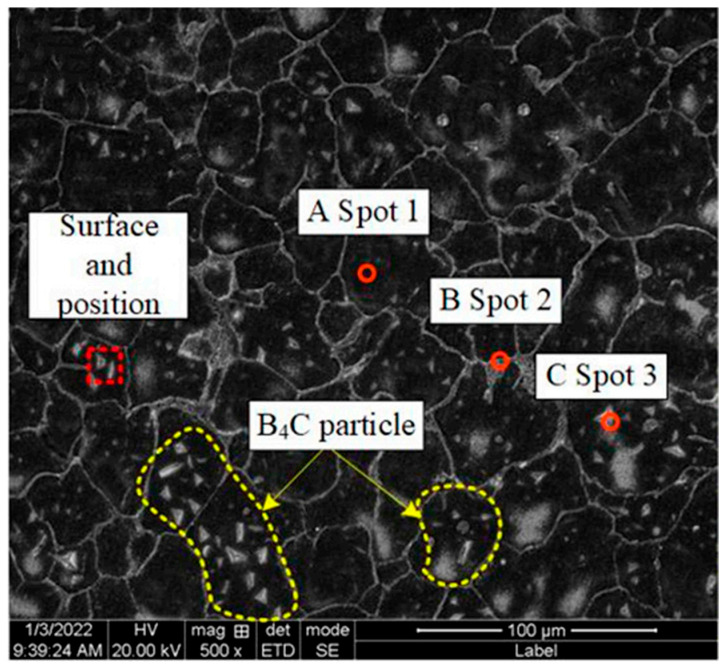
SEM images of B_4_C-reinforced WAAM 2319 aluminum alloy [69].

Hybrid reinforcement is an effective strategy for enhancing the overall performance of B4C/Al composites. The transverse tensile stress–strain curve of the WAAM 2319 aluminum alloy and the B_4_C-reinforced composite (Figure 5) shows that the addition of B_4_C significantly improves the tensile strength of the alloy. The tensile strength of the composite material is significantly higher than that of the monolithic WAAM 2319 aluminum alloy, which is mainly due to the strengthening effect of B_4_C particles and the good interface bonding between particles and matrix. The SEM image of the B_4_C-reinforced WAAM 2319 aluminum alloy (see Figure 6) shows that B_4_C particles are uniformly distributed in the aluminum matrix, and there is a good bonding state between the particles and the matrix without obvious gaps or debonding. This uniform distribution and good interface bonding are important guarantees for the improvement of the tensile strength of the composite material. Raja et al. [42] engineered an Al7075 hybrid composite incorporating 9 wt.% B_4_C and 3 wt.% TiB2 reinforcements, achieving a tensile strength of 233 MPa. This performance surpassed that of composites with either reinforcement alone, demonstrating the synergistic benefits of the dual-phase reinforcement system.

#### 3.1.2. Hardness and Toughness

**Figure 7:**
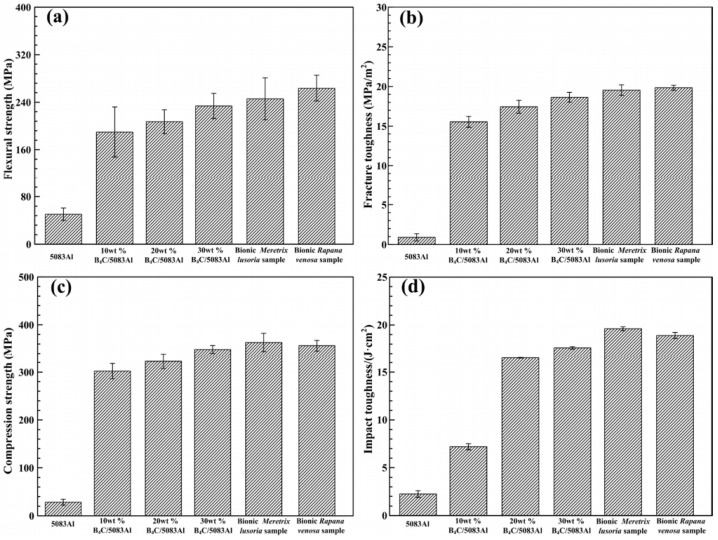
(**a**) Flexural strength; (**b**) Fracture toughness; (**c**) Compression strength and (**d**) Impact toughness values of 5083Al, B4C/5083Al composite materials, bionic Meretrix lusoria shell and bionic Rapana venosa shell layered composite materials [46].

**Figure 8:**
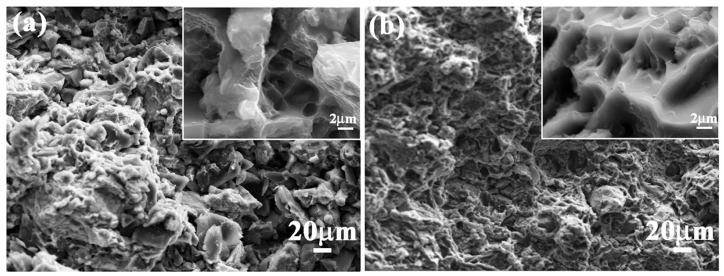
SEM images of impact fracture surfaces of B4C/5083Al composites: (**a**) inner layer of clam shell-inspired biomimetic composite, (**b**) inner layer of murex shell-inspired biomimetic composite, Reproduced with permission from [46].

The incorporation of B_4_C particles can substantially improve the hardness of the aluminum matrix. The micro-Vickers hardness distribution of the B_4_C/5083Al composite (see Figure 7) shows that the hardness of the composite with different B_4_C contents and bionic structures varies. With the increase of B_4_C content, the hardness of the composite gradually increases. Among them, the hardness of the composite with 30 wt.% B_4_C content is the highest, which is significantly higher than that of the pure 5083Al matrix. This indicates that B_4_C particles play an effective strengthening role in the composite material. Alizadeh et al. [70] observed a linear increase in composite hardness with rising B_4_C content, accompanied by a threefold improvement in wear resistance under 20 N loading conditions. Khodabakhshi et al. [61] fabricated AA8006-B_4_C nanocomposites via accumulative fold forging, achieving a record hardness of 205.4 HV for aluminum matrix composites. However, this exceptional hardness enhancement typically comes at the expense of reduced toughness, illustrating the characteristic strength–toughness trade-off in metal matrix composites. Pandey et al. [71] demonstrated that when Al_2_O_3_ content surpassed 30 wt.% and B_4_C exceeded 15 wt.%, the composite’s fracture mechanism transitioned from ductile dimple rupture to brittle cleavage fracture. To achieve an optimal balance between hardness and toughness, Thakur et al. [56] engineered an Al6063 composite co-reinforced with B_4_C and graphite. The graphite’s solid lubricating effect effectively inhibited crack propagation, thereby significantly improving the material’s fracture toughness while maintaining enhanced hardness from B_4_C. SEM images of the impact fracture surface of the B_4_C/5083Al composite (see Figure 8) show that the fracture surface of the composite with bionic clamshell and murex shell structures has a large number of ductile pits and crack bridging phenomena. These characteristics indicate that the composite has good toughness while having high hardness, which is attributed to the synergistic effect of the bionic structure design and the B_4_C particle reinforcement.

Quantitative comparisons of mechanical properties demonstrate the superiority of multi-component reinforcement systems over single-phase reinforcements. The Al7075 hybrid composite reinforced with 9% B_4_C and 3% TiB_2_ achieved a tensile strength of 233 MPa, representing a 27.3% improvement over the composite reinforced solely with 9% B_4_C (183 MPa) [42]. Similarly, the Al-3BN-0.5CNTs composite exhibited a Vickers hardness of 64 HV and a remarkable 189% increase in tensile strength compared to pure Al (31 HV) [36]. Furthermore, AA8006-B_4_C nanocomposites fabricated via the Accumulative Fold Forging (AFF) process demonstrated an exceptional hardness increase from 30 HV to 205.4 HV—a 584.7% enhancement [61]—significantly surpassing the hardness improvement achieved by the ECAP process (approximately 150%) [31].

### 3.2. Shielding Performance

Neutron shielding performance represents the most critical functional property of B_4_C/Al composites, establishing their irreplaceable role in nuclear energy applications. The material’s neutron shielding effectiveness is primarily governed by three key factors; the concentration of 10B isotopes, material thickness and incident neutron energy spectrum.

#### 3.2.1. Neutron Absorption Characteristics

**Figure 9:**
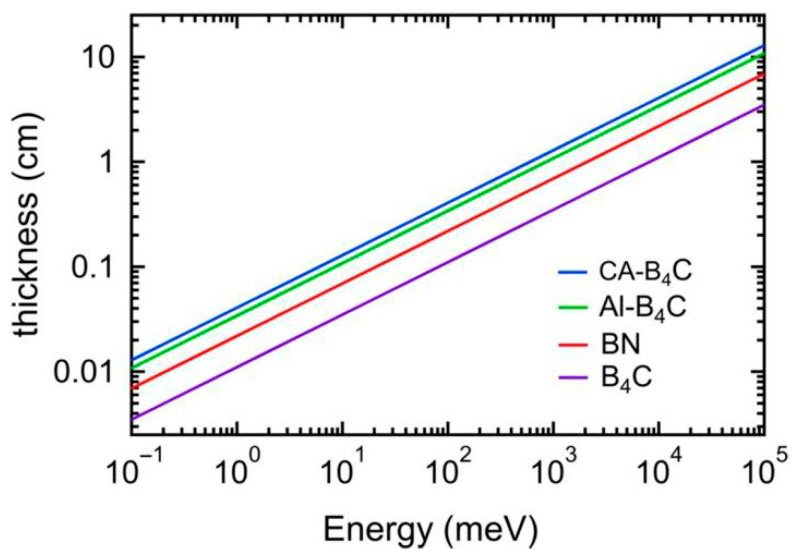
Thickness required for 99% neutron absorption in B_4_C/Al composites as a function of neutron energy [73].

Through Monte Carlo simulations, Dai et al. [39] investigated the neutron shielding performance of B_4_C/Al composites, revealing two key relationships; the neutron transmission coefficient decreased linearly with increasing B_4_C content, and it exhibited exponential decay with the increase in material thickness. The absorption fraction A of the material follows the Beer–Lambert law [72].

(1) $$A=1-\exp\left(\frac{-z}{\delta_{p}}\right)$$

(2) $$\delta p=\frac{V}{N_{f.u.}\sigma_{abs/f.u.}\varepsilon }$$

where the penetration depth can be determined in terms of the unit cell volume, *z* is the material thickness, *V* the unit cell volume, σ*_obs_* absorption cross-section, *ε* packing fraction of the material, and *N_f.u._* is the number of formula units. The material thickness required to achieve specific absorption rates is a critical design parameter. As shown in Figure 9, B_4_C/Al composites require only centimeter-scale thickness to achieve 99% neutron absorption in the thermal energy region (<1 keV), demonstrating their potential for compact shielding structures. This efficiency stems from the high ^10^B areal density (0.035–0.0389 g/cm^2^) and optimized packing fraction (90%) as calculated in Reference [73].

#### 3.2.2. Irradiation Stability

In the domain of nuclear applications, the stability of B_4_C/Al composite materials under radiation exposure is a critical performance metric. Xian et al. [34] reported that Al6061-31vol.% B_4_C composite materials prepared using the hot isostatic pressing (HIP) process at 580 °C exhibited excellent microstructural stability, with B_4_C particles uniformly distributed in the matrix and minimal interface reactions. This uniformity guarantees the stability of the material’s mechanical properties and neutron absorption performance under irradiation conditions. Quantitative measurements of the distribution of particles in the composite material demonstrated an average B_4_C content of (31 ± 0.5)%, with the standard deviation indicating optimal dispersion uniformity. This uniform distribution is critical for maintaining stable neutron shielding performance, as particle aggregation or uneven distribution may lead to localized performance degradation or reduced absorption efficiency. Furthermore, the composite material’s density is nearly equal to the theoretical value (2.642 g/cm^3^), exhibiting minimal porosity. This characteristic has been demonstrated to enhance the material’s radiation resistance and effectively suppress defect formation caused by neutron irradiation.

Quantitative analysis of neutron shielding performance reveals clear dependencies on material composition and geometry. As the B_4_C content increases from 5 wt.% to 30 wt.%, the thermal neutron (<1 keV) transmission coefficient decreases linearly from 35.2% to 7.8%, with an average reduction of 5.5 percentage points per 5 wt.% increment of B_4_C [39]. Correspondingly, when the material thickness increases from 1 cm to 5 cm, the thermal neutron absorption rate improves from 82.3% to 99.1%, following the exponential decay behavior described by the Beer-Lambert law (absorption coefficient μ = 0.72 cm^−1^) [39,73]. After neutron irradiation at a fluence of 10^18^ n/cm^2^, the Al6061-31 vol.% B_4_C composite prepared by Hot Isostatic Pressing (HIP) retained 89% of its original thermal neutron absorption cross-section, outperforming the traditionally sintered sample, which retained only 76%. This superior performance is correlated with a significantly lower vacancy cluster density (1.2 × 10^23^ m^−3^) in the HIPed material compared to the sintered counterpart (2.5 × 10^23^ m^−3^) [34].

### 3.3. Thermophysical Properties

The thermal conductivity and coefficient of thermal expansion (CTE) of B_4_C/Al composites are critical performance parameters, particularly for electronic packaging applications where efficient heat dissipation and dimensional stability are essential. Ambigai et al. [22] characterized the thermophysical properties of centrifugally cast aluminum-based B_4_C functionally graded composites (FGCs). Their study revealed that incorporating 100 μm reinforcement particles enhanced thermal conductivity by 46.4% and thermal diffusivity by 27.8%, relative to the matrix material. This improvement was attributed to the formation of more continuous thermal conduction pathways through percolation of large B_4_C particles. Nano-alumina-reinforced B_4_C/Al composites have been developed, which demonstrate exceptional thermal conductivity. These advanced materials show particular promise for defense, military, and aerospace applications where efficient heat dissipation is critical. Through first-principles calculations, Zhang et al. [37] demonstrated that SiC and phosphorus-doped graphene form strong interfacial bonds with the Al matrix, significantly enhancing the composite’s thermal conductivity, as shown in Table 6.

Quantitative comparisons of thermophysical properties highlight the influence of reinforcement size and interface engineering. In centrifugally cast Functionally Graded Composites (FGCs), the composite reinforced with 100 μm B_4_C particles achieved a thermal conductivity of 185 W/(m·K), which is 21.7% higher than that of the system reinforced with 50 μm particles (152 W/(m·K)) [22]. Conversely, the nano-B_4_C-reinforced system (5 wt.%) exhibited a reduced Coefficient of Thermal Expansion (CTE) of 8.2 × 10^−6^/K—a 28.7% decrease compared to the micron-B_4_C system (11.5 × 10^−6^/K)—but at the cost of a lower thermal conductivity of 126 W/(m·K), illustrating the characteristic trade-off between thermal conduction and dimensional stability [43]. First-principles calculations indicate that interface modification with SiC/phosphorus-doped graphene can reduce the interfacial thermal resistance from 12.5 m^2^·K/W to 6.8 m^2^·K/W, resulting in a 45.6% enhancement in thermal conductivity [37].

### 3.4. Friction and Wear Performance: Wear Mechanisms and Experimental Conditions 

#### 3.4.1. Friction and Wear Performance

**Figure 10:**
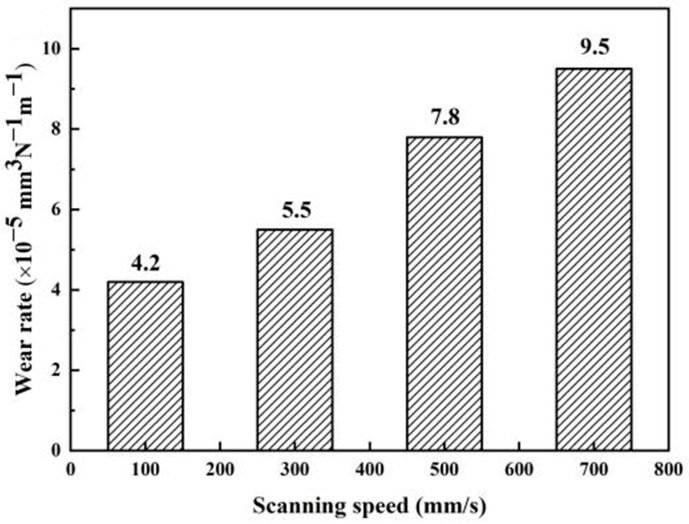
Wear rate versus scanning speed for SLM-fabricated B_4_C_p_/Al composites (B_4_C content: 20 wt.%) [54].

B_4_C/Al composites exhibit outstanding wear resistance, positioning them as promising candidates for high-performance friction components, including braking systems and bearingsThe wear rate of the SLM-fabricated B_4_C_p_/Al composite material varies with the scanning speed (see Figure 10). When the scanning speed is 100 mm/s, the wear rate of the composite material is the lowest (4.2 × 10^−5^ mm^3^/N^−1^/m^−1^). As the scanning speed increases, the wear rate shows an increasing trend. This is because the increase in scanning speed affects the forming quality and density of the composite material, thereby reducing the wear resistance. Emiru et al. [74] demonstrated that incorporating SiC, B_4_C and MoS_2_ nanoparticles into Al6061 alloy synergistically enhanced wear resistance through dual mechanisms; the solid lubrication effect of MoS_2_ and in situ formation of a protective B_2_O_3_ layer, enhanced the wear resistance of the multiphase-reinforced composite compared to the unreinforced alloy.

Ali et al. [75] identified an optimal composition in AA6351 hybrid composites, where the synergistic combination of 1 wt.% graphite and 1 wt.% B_4_C nanoparticles yielded superior tribological performance.

#### 3.4.2. Analysis of Wear Mechanisms and Experimental Conditions

##### Quantitative Influence of Experimental Conditions on Wear Performance

The quantitative effects of different experimental parameters (load, sliding speed, environment) on the wear rate of B_4_C/Al composites are summarized in Table 7 and detailed below:

**Load Effect:** When the applied load increases from 10 N to 30 N, the wear rate of a 15 wt.% B_4_C/AA6061 composite rises significantly from 2.8 × 10^−6^ mm^3^/Nm to 8.5 × 10^−6^ mm^3^/Nm, an increase of 203.6% [76]. Under high loads, the contact pressure between B_4_C particles and the counterface exceeds the yield strength of the aluminum matrix, leading to intensified plastic deformation of the matrix and enhanced abrasive cutting action. This is evidenced by an increase in the depth of plowing grooves on the worn surface from 5 μm to 18 μm, as characterized by SEM [76].

**Sliding Speed Effect:** As the sliding speed increases from 0.5 m/s to 2.0 m/s, the wear rate of a 10 wt.% B_4_C/Al composite increases from 3.2 × 10^−6^ mm^3^/Nm to 6.7 × 10^−6^ mm^3^/Nm [75]. The rising speed elevates the interfacial temperature from 50 °C to 180 °C, causing softening of the Al matrix and a consequent decrease in interfacial bonding strength. This promotes the detachment of B_4_C particles, which then act as secondary abrasives, exacerbating three-body wear.

**Environmental Medium Effect:** The wear rate of a 10 wt.% B_4_C/Al composite in a dry environment (5.1 × 10^−6^ mm^3^/Nm) is 4.25 times higher than that under oil-lubricated conditions (1.2 × 10^−6^ mm^3^/Nm) [74]. The lubricating oil forms a film approximately 2 μm thick at the friction interface, which prevents direct contact between B_4_C abrasives and the Al matrix. Simultaneously, it reduces the coefficient of friction from 0.6 to 0.2, significantly suppressing both adhesive and abrasive wear.

##### Dominant Wear Mechanisms in B_4_C/Al Composites

Based on SEM/TEM characterization and EDS analysis of worn surfaces, the wear mechanisms of B_4_C/Al composites can be categorized into four types, strongly correlated with material composition and experimental conditions:

**Abrasive Wear (Dominant Mechanism):** Detachment of B_4_C particles due to interfacial bonding failure forms hard abrasives 5–20 μm in diameter. These particles cause cutting and plowing of the Al matrix during friction, manifesting as grooves (2–20 μm deep) parallel to the sliding direction and the accumulation of wear debris [76]. As the B_4_C content increases from 5 wt.% to 15 wt.%, the contribution of abrasive wear rises from 45% to 70%. However, the “load-bearing” effect of the B_4_C particles partially counteracts the wear, resulting in an overall 67.1% decrease in the total wear rate [76].

**Adhesive Wear:** In systems with low B_4_C content (<5 wt.%), the large exposed area of the Al matrix facilitates adhesive transfer to the counterface (e.g., steel). The worn surface exhibits adhesive pits 10–30 μm in diameter and tear marks, accompanied by a high wear rate of 8.5 × 10^−6^ mm^3^/Nm [75]. Adding 0.5 wt.% CNTs reduces the proportion of adhesive wear from 35% to 12% by enhancing the interfacial strength and minimizing matrix adhesion [36].

**Oxidative Wear:** At sliding speeds > 1.5 m/s or ambient temperatures > 150 °C, a rapid-forming Al_2_O_3_ oxide film (50–100 nm thick) develops on the Al matrix surface. Initially, this film can reduce the wear rate by approximately 20%. However, due to its brittleness, the film is prone to fracture, and the resulting oxide fragments become new abrasives, leading to wear rate fluctuations of up to 15% [74].

**Synergistic Wear (Hybrid-Reinforced Systems):** In hybrid systems like Al-3BN-0.5CNTs/B_4_C, the solid lubricating effect of h-BN (reducing the coefficient of friction from 0.6 to 0.25) suppresses adhesive wear, while the “crack-bridging” effect of CNTs reduces the detachment of B_4_C particles. This synergy decreases the contribution of abrasive wear from 70% to 40% and results in an overall wear rate that is 31.0% lower than that of single B_4_C-reinforced systems [36]. SEM observations confirm that the hybrid system’s worn surface exhibits shallow grooves (~3 μm deep) and no significant adhesive marks [36].

##### Strategies for Controlling Wear Mechanisms

Based on the above mechanistic analysis, the wear performance of B_4_C/Al composites can be optimized through the following strategies:

**Interface Strengthening to Suppress Abrasive Wear:** Applying a nano-SiO_2_ coating to B_4_C particles increases the interfacial bonding strength from 60 MPa to 95 MPa, reduces the B_4_C particle detachment rate by 40%, and decreases the depth of grooves caused by abrasive wear to below 5 μm [77].

**Incorporation of Solid Lubricants to Mitigate Adhesive Wear:** Adding 1–3 wt.% h-BN or graphite facilitates the formation of a continuous lubricating film at the friction interface. This reduces the proportion of adhesive wear to below 10% and concurrently lowers the wear rate by 30–40% [36,56].

**Process Optimization to Inhibit Oxidative Wear:** Controlling the sliding speed to below 1.0 m/s or employing an oil-lubricated environment keeps the interface temperature below 100 °C, preventing the fracture of the Al_2_O_3_ film and reducing wear rate fluctuations from 15% to 5% [74,75].

**Synergistic Control via Multi-scale Reinforcement:** Utilizing a hybrid reinforcement strategy of “15 wt.% micron B_4_C + 0.5 wt.% nano CNTs” leverages the combined benefits of B_4_C’s anti-wear properties and CNTs’ interface strengthening effect. This approach stabilizes the wear rate below 1.9 × 10^−6^ mm^3^/Nm, meeting the requirements for friction components in aerospace applications [36].

Quantitative evaluation of tribological performance reveals the effects of reinforcement content and operational parameters. As the B_4_C content in AA6061-B_4_C composites increases from 5 wt.% to 15 wt.%, the wear rate under a 20 N load and 1 m/s sliding speed decreases from 8.5 × 10^−6^ mm^3^/Nm to 2.8 × 10^−6^ mm^3^/Nm, a reduction of 67.1%. A further increase to 20 wt.% B_4_C only marginally reduces the wear rate to 2.5 × 10^−6^ mm^3^/Nm, indicating a diminishing returns effect [76]. The wear rate of the 15 wt.% B_4_C composite increases by 203.6%, from 2.8 × 10^−6^ mm^3^/Nm at 10 N load to 8.5 × 10^−6^ mm^3^/Nm at 30 N load [76]. Similarly, increasing the sliding speed from 0.5 m/s to 2.0 m/s raises the wear rate from 3.2 × 10^−6^ mm^3^/Nm to 6.7 × 10^−6^ mm^3^/Nm [75]. Notably, the hybrid Al-3BN-0.5CNTs system exhibits a wear rate of 1.9 × 10^−6^ mm^3^/Nm, which is 31.0% lower than that of the single 15 wt.% B_4_C-reinforced system [36].

### 3.5. Other Performances

#### 3.5.1. Corrosion Resistance

Han et al. [33] conducted a systematic investigation of the electrochemical behavior of B_4_C/Al composites, revealing an inverse correlation between B_4_C volume fraction and corrosion resistance. This degradation was attributed to two primary mechanisms; galvanic coupling between the aluminum matrix and B_4_C particles, and an oxygen diffusion-controlled corrosion process at the reinforcement-matrix interfaces. Huang et al. [78] used low-pressure cold spraying technology to create an Al-30% B_4_C composite coating on a 5083 aluminum alloy substrate. Friction and wear tests revealed that the coating’s wear rate was one-third that of the base aluminum alloy. However, electrochemical tests showed that the coating’s corrosion rate was slightly higher than that of the base material. The study revealed that the unique fragmentation and embedding behavior of B_4_C particles during deposition significantly influenced the coating’s microstructure, thereby determining its neutron absorption performance and wear- and corrosion-resistant properties. The residual content of B_4_C particles is a key factor in the coating’s functional properties.

#### 3.5.2. Processing Performance

The processing characteristics of B_4_C/Al composites critically influences their industrial applicability. Mohankumar et al. [62] developed a predictive model for water-jet cutting angles in Al6063-B_4_C composites, effectively addressing dimensional precision challenges in machining hard particle-reinforced lightweight alloys. Kumaran et al. [79] achieved significant improvements in processing quality through parameter optimization of pulsed: YAG laser machining for Al6351-B_4_C composites. Table 8 comprehensively summarizes the key performance metrics and critical influencing factors for typical B_4_C/Al composite systems. The optimization of machining parameters, including cutting speed, feed rate, and cutting depth, has been demonstrated to reduce surface roughness and energy consumption during the milling process of aluminum-based composite materials (AMCs) [80]. A thrust prediction model during drilling is imperative for the optimization of machining such composites [81]. The utilization of acoustic emission technology in the context of monitoring wire electrical discharge machining (WEDM) has been demonstrated to yield valuable Reference information for the machining process [82]. In the domain of laser processing, attaining optimal surface quality necessitates meticulous selection of processing parameters [83].

### 3.6. Quantitative Analysis of Property-Process Relationships

Based on the comprehensive data of fabrication processes and corresponding properties, quantitative “process-property” relationships for B_4_C/Al composites are established as follows:

Influence of Fabrication Process on Density: The achieved relative density is highly process-dependent, ranking as: Hot Isostatic Pressing (HIP) (99.6%) > Spark Plasma Sintering (SPS) (98.7%) > Stir Casting (92.5%) > Conventional Sintering (91.9%). A strong positive correlation is observed, where an increase of 1% in relative density corresponds to an average tensile strength improvement of approximately 12 MPa [38,40].

Effect of B_4_C Content on Properties: Increasing the B_4_C content from 5 wt.% to 15 wt.% in stir-cast composites results in a 73.3% enhancement in hardness (from 75.2 HRB to 130.3 HRB). Conversely, this leads to a significant 63.1% reduction in elongation, decreasing from 42.6% to 15.7% [21], highlighting the typical strength-ductility trade-off.

Scale Effect of Reinforcement Phase: Composites reinforced with nano-sized B_4_C particles achieve a compressive strength of 1065 MPa, which is 3.5 times greater than that of composites reinforced with micron-sized B_4_C particles (301 MPa). This remarkable enhancement is primarily attributed to the more potent strengthening mechanisms afforded by nanoparticles, namely Orowan strengthening and the Hall-Petch grain refinement effect [43].

## 4. Application Fields

B_4_C/Al composites have been successfully implemented in multiple high-tech fields including nuclear energy, defense and military systems, and aerospace due to their exceptional multifunctional performance, high mechanical properties (such as specific strength, hardness, and wear resistance), excellent neutron shielding capabilities, superior thermophysical properties (e.g., thermal conductivity and tunable coefficient of thermal expansion), and lightweight characteristics. Their application scope continues to expand into emerging technological domains, advanced electronic packaging, high-performance transportation components, and specialized tool manufacturing. This section details their principal industrial applications, along with representative case studies.

### 4.1. Nuclear Energy Engineering

In nuclear energy applications, B_4_C/Al composites serve as critical neutron absorbing materials, particularly in spent fuel storage systems and reactor shielding components. These composites offer three key advantages, including an exceptionally high neutron absorption cross-section, superior thermal conductivity and optimal mechanical strength for structural applications.

#### 4.1.1. Spent Fuel Storage

**Figure 11:**
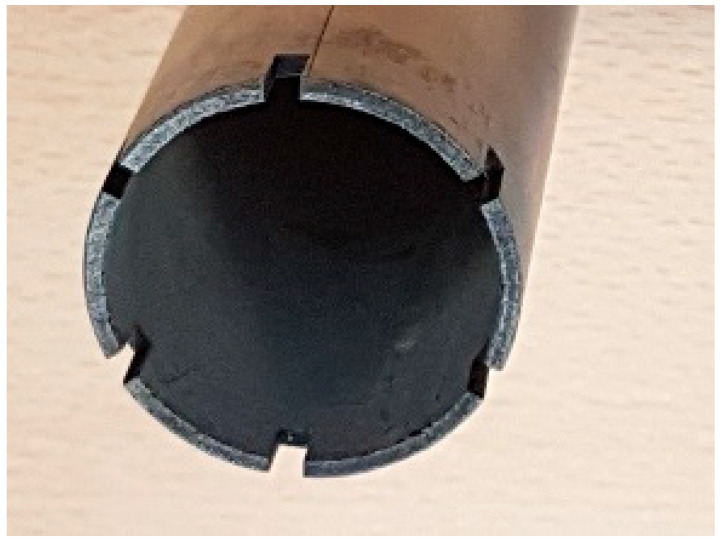
Fracture morphology of the annular component (Φ45 mm) fabricated from Al-W-B composite recycled material [85].

Spent fuel storage stands as the most prominent and well-established application of B_4_C/Al composites. The B_4_C/Al nuclear waste storage system incorporates two key design innovations; a reinforced auxiliary structure with moisture-resistant treatment, and synergistic integration of reinforced boron (high 10B neutron absorption cross-section) and tungsten-nickel-iron alloy (excellent γ-ray shielding). This optimized configuration enables radiation reduction, long-term stability of the shielding layer and environmental resilience. This property can prevent the absorption plate from brittle failure under dynamic loads (e.g., seismic activities) during the storage of spent fuel. Shirvanimoghaddam et al. [84] fabricated B_4_C/Al neutron absorption plates for nuclear fuel assemblies using the stir casting method. The fracture morphology of the annular component fabricated from Al-W-B composite recycled material (see Figure 11) shows a mixed ductile-brittle fracture mode. The presence of fine ductile pits on the fracture surface indicates that the material has a certain degree of toughness, which can effectively avoid brittle failure under dynamic loads during spent fuel storage, ensuring the safety and reliability of the material in service. They optimized the casting temperature to improve the mechanical properties of the neutron absorption plates.

#### 4.1.2. Reactor Shielding

Aluminum-based boron carbide (B_4_C/Al) composite materials have been demonstrated to have unique application value in reactor radiation shielding. This is due to their high-efficiency neutron absorption capability and comprehensive advantages in radiation safety.

With regard to the optimization of shielding performance, the neutron shielding effect of B_4_C/Al composite materials is closely related to material composition and thickness. Dai et al. [39] discovered that the neutron transmission coefficient of B_4_C/Al composite materials decreases linearly with increasing boron carbide content and exponentially with increasing material thickness through Monte Carlo simulations. Furthermore, the samples demonstrated heightened sensitivity in terms of energy response characteristics within the thermal neutron energy range. This superior performance in shielding is a notable advantage over conventional shielding materials.

In the context of irradiation safety verification, ensuring the stability of the material under reactor irradiation conditions is of paramount importance. Mi et al. [44] conducted a thermal safety analysis using computational fluid dynamics (CFD) simulations to verify the structural integrity and thermal stability of B_4_C/Al composite materials under in-core irradiation conditions. This analysis ensured the safety of the materials as reactor shielding materials.

### 4.2. National Defense and Military Industry

Boron carbide (B_4_C)-reinforced aluminum matrix composites have emerged as an ideal new-generation armor material due to their high hardness, low density and excellent ballistic resistance. The aluminum matrix provides lightweight characteristics, while the B_4_C reinforcement phase endows the material with outstanding impact resistance capabilities.

**Figure 12:**
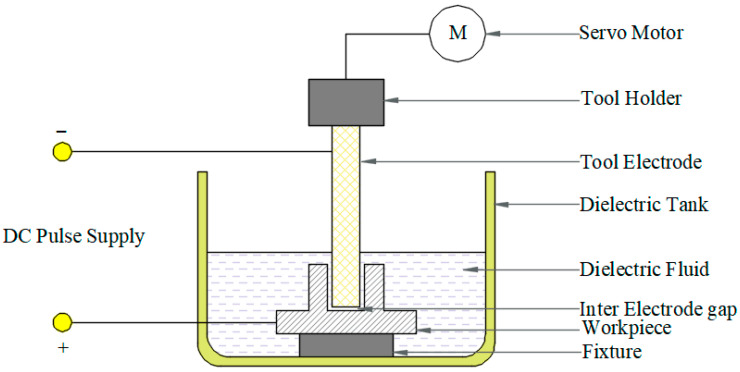
Arrangement and components of an electrical discharge machining (EDM) setup [62].

**Figure 13:**
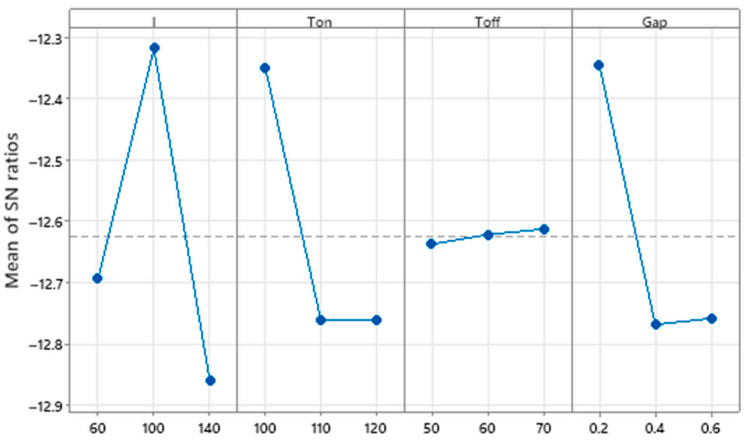
Main effect plot for surface roughness of B_4_C-reinforced Al7075 composite [62].

Boron carbide has unique advantages in armor applications, with exceptional comprehensive properties such as extremely high hardness (Mohs hardness 9.3), low density (2.52 g/cm^3^), and excellent thermochemical stability [5]. These characteristics establish B_4_C as an indispensable material for advanced armor systems. Pul et al. [20] systematically investigated the ballistic performance of 7075 aluminum alloy composites reinforced with SiC and B_4_C particles. Figure 12 shows the schematic diagram, photograph and orientation diagram of the processed B_4_C-reinforced Al7075 composite samples. The reasonable design of the sample shape and orientation is conducive to accurately testing the mechanical properties of the composite material in different directions, and provides a reliable basis for evaluating the ballistic performance of the armor material. Their study demonstrated that increasing the reinforcement ratio progressively improves ballistic resistance, with an optimal 20% reinforcement content achieving the ideal balance between ballistic protection and machinability. They quantitatively mapped the size distribution and spatial arrangement of these mesoscale defects, establishing a critical foundation for optimizing armor material properties through defect engineering. The main effect plot for surface roughness of B_4_C-reinforced Al7075 composite (see Figure 13) shows that process parameters such as current and electrode gap have significant effects on the surface roughness of the composite material. Furthermore, micro-defects introduced during processing, such as pores and agglomerates, can also affect the consistency of the material’s ballistic performance. The application of three-dimensional non-destructive characterization techniques like micro-computed tomography (micro-CT), as described by Moorehead et al. [86], enables the quantitative assessment of internal defects within composites, providing critical data support for process optimization and performance prediction. By optimizing these parameters, the surface roughness of the composite material can be effectively controlled, which is of great significance for improving the ballistic performance of the armor material

Zhang et al. [53] engineered three-modal B_4_C/Al composites featuring amorphous multilayer interfaces, which demonstrated significantly improved impact resistance showing 40% increase in fracture toughness and 30% higher energy absorption compared to conventional composites under ballistic testing. Kumar et al. [87] demonstrated that garnet and B_4_C-reinforced aluminum matrix composites exhibit superior tensile strength and hardness, while significantly reducing the wear rate properties, making them highly suitable for high-stress military vehicle transmission components.

### 4.3. Aerospace

**Figure 14:**
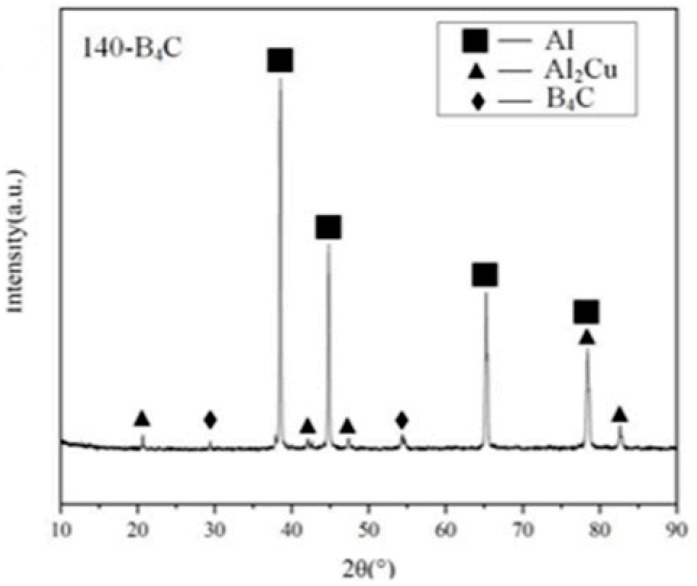
XRD patterns of B_4_C-reinforced WAAM 2319 aluminum alloy (deposition speed: 140 mm/min) [69].

B_4_C/Al composites are ideal for aerospace due to their lightweight, high strength, and excellent high-temperature resistance. Their low density, reinforced stiffness, and thermal stability make them suitable for aircraft and spacecraft components. The h-BN and CNTs co-reinforced aluminum matrix composites developed by Khan et al. [36] exhibit outstanding properties, achieving a high relative density of 97.7%. The XRD pattern of the B_4_C-reinforced WAAM 2319 aluminum alloy (see Figure 14) shows that the main phases of the composite material are Al, Cu and B_4_C, and there are no other impurity phases. This indicates that the composite material has good phase stability, and no abnormal phase transition occurs during the preparation process, which ensures the stable performance of the composite material in aerospace applications

Notably, for B_4_C/Al composites fabricated via selective laser melting (SLM), the relative densification rate decreases from 97.1% to 85.0% as the scanning speed increases from 100 mm/s to 700 mm/s—this is due to insufficient molten pool temperature and powder melting at high scanning speeds [54]. Combined with the high densification of BN-CNTs/Al (97.7% relative density) via sintering, these results confirm that both reinforcement synergy and process parameters (e.g., SLM scanning speed) govern the density and porosity of Al-based composites. A quantitative comparison of the densification behavior achieved in these multi-component systems, underlining the synergy between reinforcement composition and processing parameters, is provided in Table 9. Their superior strength-to-weight ratio and enhanced mechanical performance position them as ideal candidates for high-strength, lightweight structural materials in the aerospace industry. Ma et al. [88] emphasized that particle-reinforced Al matrix composites offer significant advantages for aerospace lightweight applications. Their research highlights that the size and spatial distribution of both matrix grains and reinforcing particles play a crucial role in determining the material’s mechanical properties, making these composites highly suitable for high- performance aerospace structures. Through optimized friction stir welding process parameters, Jamaludeen [89] achieved a significant reduction in wear rate to 154.21 × 10^−5^ mm^3^/m for AA6092/B_4_C composites, substantially improving their reliability for aviation applications. Sekhar et al. [45] developed a sustainable high-performance composite by reinforcing recycled aluminum can matrix with granite particles and B_4_C. The material exhibited exceptional, mechanical properties, achieving compressive strength of 1124 MPa along with 28% enhanced wear resistance, successfully combining resource recycling with superior performance. Ambigai et al. [22] developed a centrifugally cast Al-based B_4_C FGCs with remarkable thermomechanical properties. Their study revealed that a 50 μm reinforcing phase improved the tensile strength by 31%, while the 100 μm reinforcing phase enhanced the thermal conductivity by 46.4% and the thermal diffusivity by 27.8%. These tailorable properties make the composite ideal for aerospace thermal management systems, particularly for graded heat exchangers and heat sinks accommodating variable thermal loads.

### 4.4. Transportation

B_4_C/Al composites are highly competitive for automotive and rail transport applications due to their optimal combination of lightweight properties, excellent wear resistance, and cost efficiency. The low-density aluminum matrix enables significant weight reduction, while the B_4_C reinforcement enhances surface durability. These advantages, along with competitive manufacturing costs, make B_4_C/Al composites particularly suitable for high-performance moving components where reducing mass and extending service life are critical requirements.

#### 4.4.1. Automobile Parts

Chandel et al. [90] demonstrated that Al matrix composites incorporating both hard ceramic particles and soft reinforcements achieve optimal performance-significantly enhancing strength, hardness and wear resistance while reducing brittleness. This balanced approach effectively meets automotive industry requirements for lightweight materials with high strength and superior wear resistance. Thakur et al. [56] developed an Al6063-B_4_C-Gr hybrid composite with optimized reinforcement ratios, demonstrating superior wear resistance for high-speed applications. Madhu et al. [27] innovatively incorporated rubber ash and B_4_C in varying ratios into Al-based FGCs, achieving both cost reduction and enhanced tensile strength through synergistic reinforcement-offering a novel solution for automotive lightweight applications. Aabid et al. [91] enhanced the dry sliding wear performance of aluminum hybrid composites, offering potential for developing lubricant-reduced automotive braking systems.

#### 4.4.2. Rail Transit

Venkatesan et al. [92] developed an optimized die contour technology combining cosine and cubic polynomial profiles, which significantly reduced extrusion loads while ensuring uniform metal flow in B_4_C/Al composites. This advancement provides an efficient new manufacturing process for producing large structural components in rail transit applications.

### 4.5. Electronics Industry

**Figure 15:**
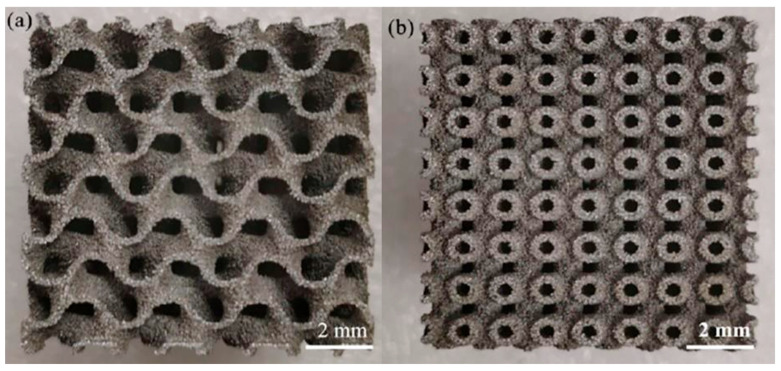
Two representative components (**a**,**b**) with complex matrix structures fabricated by SLM [54].

In electronics industry, B_4_C/Al composites have become essential materials for electronic packaging and thermal management systems, owing to their tunable CTE and superior thermal conductivity. These tailored thermophysical properties enable optimal heat dissipation while minimizing thermal stresses in sensitive electronic components. Ziani et al. [68] developed an Al/Mo/SiC tri-component periodic multilayer film exhibiting high EUV reflectivity (48% at 17.3 nm), which has been successfully implemented in the solar orbiter mission’s EUV imager. Figure 15 shows two representative complex structural components fabricated by SLM using B_4_C particles and Al powder as raw materials. The components have precise structural dimensions and good surface quality, which shows that SLM technology has unique advantages in manufacturing complex structural Al-based composite components for electronic packaging. The uniform dispersion of B_4_C particles in the components ensures the stable performance of electronic devicesZhang et al. [37] demonstrated that strong interfacial bonding between SiC/P-doped graphene and Al matrix can simultaneously enhance the composite’s mechanical strength and thermal performance, offering a novel design strategy for advanced electronic packaging materials. The laser surface treatment of B_4_C/Al composites by Yilbas et al. [65] significantly enhanced both surface microhardness and hydrophobicity through the formation of a dense sub-micron grain layer containing AlN compounds, making these modified composites particularly suitable for electronic device enclosure applications. Alattar et al. [93] demonstrated that incorporating 5% B_4_C particles into Al matrix composites yields a uniform fine-grained microstructure, optimizing both hardness and ultimate tensile strength, properties particularly advantageous for heat-sensitive electronic components requiring efficient thermal dissipation.

### 4.6. Other Applications

**Figure 16:**
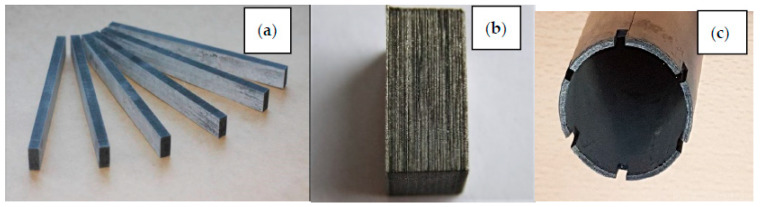
Elements for grinding and polishing devices: honing stones 6 × 10 × 120 mm (**a**,**b**); ring drill Φ45 mm (**c**) [85].

Beyond these primary applications, B_4_C/Al composites are also utilized in specialized domains such as tool manufacturing and sports equipment. Mironovs et al. [85] developed a powder metallurgy recycling process for aluminum-based B-W fiber composites, enabling the reuse of high-strength fiber waste in cutting tool manufacturing. Figure 16. Cutting inserts (model: CNMG 120408) made from recycled B-W fiber-reinforced Al matrix composites [85]. The inserts are sintered from recycled composite powder (15 wt.% B-W fibers) at 650 °C under 40 MPa, then precision-ground to meet cutting tool geometry standards. Their surface Vickers hardness reaches 185 HV—twice that of traditional high-speed steel inserts—resulting in a low wear rate of 0.02 mm^3^/min when cutting 45# steel (150 m/min cutting speed). This application not only realizes the recycling of B-W fiber composite waste (reducing material costs by ≈30%) but also provides a high-wear-resistance alternative for industrial cutting tools, expanding the practical use of B_4_C/Al composites beyond high-tech fields to general manufacturing. Panneerselvam et al. [94] demonstrated that AA6063-B_4_C-ZrSiO_4_ hybrid composites exhibit superior wear resistance and a reduced coefficient of friction in tribological tests, rendering them ideal as high-performance sports equipment materials. Table 10 summarizes the key performance metrics and representative applications of B_4_C/Al composites across different application fields.

## 5. Summary and Outlook

After decades of development, significant advancements of B_4_C/Al composites have been achieved in processing techniques, performance optimization, and engineering applications, yet critical challenges remain. This section summarizes current research milestones, analyzes persistent limitations, and outlines future development priorities.

### 5.1. Summary of Research Progress

From a material system perspective, B_4_C/Al composites have progressed from simple binary formulations to advanced multi-component hybrid architectures. Khan et al. [36] developed an Al matrix composite co-reinforced with h-BN and CNTs, achieving 97.7% theoretical density while significantly enhancing mechanical properties. Raja et al. [42] developed a multi-scale Al7075 composite reinforced with hybrid B_4_C-TiB_2_ particles, achieving optimal comprehensive performance. This multi-component design strategy has emerged as a critical approach for advanced material optimization. Regarding fabrication methods, conventional processing techniques continue to undergo refinement while novel approaches are rapidly advancing. Chen et al. [15] optimized the HIP process for manufacturing large-scale B_4_C/Al composite plates. Sun et al. [63,64] were the first to develop additive manufacturing (AM) technology, which facilitates the economical and precise fabrication of aluminum-based composite structures. The implementation of wire powder arc additive manufacturing (WPA-AM) methodologies enabled the effective incorporation of ceramic particles, including B_4_C, SiC, TiC, and WC/W_2_C, into the aluminum matrix. Through the optimization of process parameters, a substantial enhancement in the mechanical properties of the material was attained. The following text is intended to provide a comprehensive overview of the subject matter. Khodabakhshi et al. [61] pioneered an innovative accumulative fold-forging process to fabricate ultrafine-grained nanocomposites. This breakthrough in processing technology establishes a robust foundation for enhancing material properties. Performance investigations have progressively advanced from macroscopic mechanical characterization to comprehensive understanding of microscopic deformation mechanisms. Li et al. [43] elucidated the synergistic reinforcement mechanisms in Al-based composites, demonstrating enhanced performance through multi-scale strengthening effects. Dai et al. [39] established quantitative composition-performance relationships for radiation shielding applications. Collectively, these studies provide fundamental theoretical guidance for advanced material design. The engineering applications of these composites have significantly expanded beyond traditional nuclear shielding to emerging fields including armor protection systems and high-performance electronic packaging, reflecting their versatile property portfolio. The diverse applications of B_4_C/Al composites, including nuclear waste storage tanks [95], high-performance armor systems [20] and EUV optical components [68] demonstrate their exceptional versatility across multiple engineering domains [75].

### 5.2. Key Issues

Despite significant advancements, critical challenges still exist for B_4_C/Al composites.

#### 5.2.1. Interface Response Control

Interface control remains a critical research challenge in B_4_C/Al composites. Excessive formation of brittle phases at the B_4_C/Al interface significantly reduces both fracture toughness and plastic deformation capacity. However, systematic methodologies for precisely regulating interfacial reaction products through process optimization remain largely underdeveloped. Han et al. [33] demonstrated that higher B_4_C content exacerbates galvanic corrosion in B_4_C/Al composites, revealing the critical role of interface stability in corrosion resistance [66]. Future research must address three key priorities; elucidating the thermodynamic and kinetic mechanisms of interfacial reactions, establishing quantitative structure-property relationships at interfaces, and developing universal interface engineering strategies.

#### 5.2.2. Process Repeatability

Process repeatability remains the critical bottleneck limiting large-scale industrial adoption. As Bhowmik et al. [35] demonstrated, both controlled and uncontrolled variables in stir casting processes significantly impact final product quality. However, comprehensive quantitative models correlating process parameters with material properties remain underdeveloped [62]. Butola et al. [59] optimized FSP parameters using response surface methodology, demonstrating that theory-guided process design is essential for ensuring material property consistency. To achieve precise process control, future efforts should focus on developing real-time monitoring and intelligent control systems.

#### 5.2.3. Irradiation Damage Mechanism

The limited understanding of irradiation damage mechanisms remains a critical barrier for nuclear applications. The long-term irradiation effects on microstructure evolution and performance degradation require further investigation [96]. Although the graphitized nanocarbon helium-absorption method [37] demonstrates effectiveness, a more comprehensive irradiation damage mitigation strategy is still required. Future research should integrate advanced characterization techniques with multiscale simulations to develop predictive irradiation damage models.

#### 5.2.4. Research Gap in Wear Mechanisms

Current understanding of the wear mechanisms under multi-factor coupling remains insufficient. Under extreme conditions (e.g., temperature > 500 °C, neutron fluence > 10^18^ n/cm^2^), the quantitative threshold for the transition of the dominant wear mechanism in B_4_C/Al composites from “abrasive-dominated” to “oxidative-adhesive synergistic” has not been established. Furthermore, a quantitative model correlating the wear rate with microstructural evolution—such as the distribution of B_4_C particles and the composition of interfacial phases—is still lacking. This knowledge gap significantly hinders the application of these composites in high-end scenarios, such as friction components in nuclear reactors or bearings in aero-engines. Future research should employ In Situ tribo-characterization techniques (e.g., In Situ SEM tribometry) to elucidate the transition laws of wear mechanisms under extreme service conditions.

### 5.3. Future Development Direction

To address these challenges, future research on B_4_C/Al composites can focus on the following key directions.

#### 5.3.1. Multi-Scale Interface Design

Future research can develop multiscale interface design methodologies to precisely control interfacial performance in B_4_C/Al composites. By integrating atomic-scale first-principles calculations [37], nanoscale interface engineering, and macroscale alloy design, this hierarchical approach enables the construction of gradient functionalized interfaces that simultaneously enhance strength, fracture toughness and environmental stability. Critical implementation strategies will leverage surface modification, nano-coating technologies [77] and precision microalloying to optimize reinforcement-matrix compatibility and bulk property uniformity.

#### 5.3.2. Intelligent Preparation Process

Future study can focus on developing intelligent manufacturing processes to improve property consistency and repeatability in B_4_C/Al composites. By integrating machine learning and digital twin technologies, a quantitative process-structure-property prediction model can be established, enabling real-time process optimization [97]. As demonstrated by Karimi et al. [98] through automated grain analysis in WAAM, applying machine learning to AM process data is a vital step toward building such predictive models. Advanced digital manufacturing techniques, including AM and cold spraying, will play increasingly critical roles in achieving these objectives.

#### 5.3.3. Prediction of Extreme Environmental Behaviors

To enhance the prediction and evaluation of material behavior in extreme environments, future research should focus on developing multi-physics coupled irradiation damage models. By integrating artificial intelligence with these models, one can accurately predict material performance evolution under complex conditions, including long-term irradiation, high-temperature, and high-pressure exposures, thereby providing reliable guidance for engineering applications [99]. In situ characterization techniques and accelerated testing methodologies will be critical to validating these predictive frameworks.

#### 5.3.4. Sustainable Development

Future work on Al-based composites must prioritize sustainable development and closed-loop material cycles. As demonstrated by Mironovs et al. [85], developing energy-efficient fabrication processes with minimal emissions, along with establishing robust recycling protocols, will be critical for reducing environmental impact [100]. These efforts should be complemented by advanced design strategies such as functional gradient architectures and structure-function integration, which optimize material utilization efficiency across multiple lifecycles. The integration of these approaches combining green manufacturing techniques, effective end of life recovery methods, and intelligent material design represents a comprehensive pathway toward sustainable composite technologies.

## Figures and Tables

**Table 1:** Quantitative Performance Comparison of B_4_C/Al Composites Fabricated by Different Powder Metallurgy Processes.

Process	B_4_C Content (wt.%)	Relative Density (%)	Tensile Strength (MPa)	Microhardness (HV)	Reference
Conventional Vacuum Sintering	30	91.9	86	52	[38]
Semi-solid HIP	30	99.6	301	128	[38]
Microwave Sintering	10	92.3	110	61.5	[40]
SPS	10	98.7	235	189.3	[40]

**Table 2:** Control factors and level values for EDM of Al7075/B_4_C composites [62].

Parameter	Level 1	Level 2	Level 3
Current (I, A)	60	100	140
Pulse ON Time (Ton, ms)	100	110	120
Pulse OFF Time (Toff, ms)	50	60	70
Electrode Gap (Gap, mm)	0.2	0.4	0.6

**Table 3:** Comparison of fabrication methods for B_4_C/Al composites.

Fabrication Method	Process Characteristics	B_4_C Content (wt.%)	Typical Properties	Reference
HIP	Densification under high temperature/pressure; uniform reinforcement distribution	10–35	Tensile strength > 300 MPa; elongation > 3%	[38]
SPS	Rapid sintering process; refined grain structure	10–20	High relative density; significantly enhanced microhardness	[40]
Stir Casting	Low-cost; suitable for mass production	5–15	Hardness increases with B_4_C content	[35]
ECAP	Significant grain refinement; improved mechanical properties	5–15	Enhanced hardness and wear resistance with increasing passes	[31]

**Table 4:** Cost structure comparison of AM and ECAP processes for B_4_C/Al composites fabrication.

Process Type	Equipment Investment (kUSD)	Material Cost (USD/kg)	Energy Cost (USD/kg)	Unit Production Cost (USD/kg)	Suitable Production Scale	References
ECAP	110–165	6.9–11	0.35	0.7–1.4	Mass Production (>100 tons/yr)	[31]
LPBF (AM)	415–690	110–138	6.9–11	28–69	Small Batch (<5 tons/yr)	[41,64]
WPA-AM (AM)	207–276	83–110	5.5–8.3	17–25	Medium-Small Batch (5–20 tons/yr)	[41]

Note: Cost conversions from CNY to USD are approximate, using an indicative exchange rate for comparative purposes.

**Table 5:** Summary of Fabrication-Property-Application Relationships for B_4_C/Al Composites.

Fabrication Method	Process Characteristics	Typical B_4_C Content (wt.%)	Key Properties	Typical Applications	References
Powder Metallurgy (HIP)	High temp/pressure; liquid-phase sintering & densification	30	High relative density (99.6%), Tensile strength (301 MPa)	Nuclear shielding, Spent fuel storage containers	[38]
Spark Plasma Sintering (SPS)	Rapid sintering; Plasma activation	10	High microhardness (189.3 HV), High relative density (98.7%)	Armor plates, Personal protection	[40]
Stir Casting	Low cost, mass production; Parameter sensitivity	15	Increased hardness with content; Tensile strength (203 MPa)	Automotive brake rotors, Structural supports	[35,55]
Melt Infiltration	Suitable for high volume fractions; Uniform distribution	>20	High density (96.8%), Good tensile strength (267 MPa)	High-performance neutron absorbers	[57]
ECAP/FSP	Severe plastic deformation; Grain refinement	5–15	Significantly enhanced hardness and wear resistance	Aerospace wear-resistant components	[31,59]
Additive Manufacturing (AM)	Complex geometry formation; Multi-material/Gradient design capability	5–15	High strength (320 MPa) with good ductility	Aerospace lightweight structures, Custom functional parts	[63,64]
Surface Composite Technology	Substrate surface modification; Cost-effective	5–15	Enhanced surface hardness, wear and corrosion resistance	Electronic packaging, Space optical components	[65,67]

**Table 6:** Electronic configuration and radius cut-off for the elements used in this study [37].

Element	Electron Configuration	Radius Cut-Off (Bohr)
Aluminum (Al)	3s^2^ 3p^1^	1.90
Carbon (C)	2s^2^ 2p^2^	1.51
Silicon (Si)	3s^2^ 3p^2^	1.91
Phosphorus (P)	3s^2^ 3p^3^	1.91
Boron (B)	2s^2^ 2p^1^	1.70
Nitrogen (N)	2s^2^ 2p^3^	1.20

**Table 7:** Influence of Experimental Conditions on the Wear Rate of B_4_C/Al Composites.

B_4_C Content (wt.%)	Load (N)	Sliding Speed (m/s)	Condition	Wear Rate (×10^−6^ mm^3^/Nm)	Interface Temperature (°C)	Reference
15	10	1.0	Dry	2.8	50	[76]
15	30	1.0	Dry	8.5	95	[76]
10	20	0.5	Dry	3.2	42	[75]
10	20	2.0	Dry	6.7	180	[75]
10	20	1.0	Oil lubrication	1.2	35	[74]

**Table 8:** Typical Properties and Influencing Factors of B_4_C-Reinforced Aluminum Matrix Composites.

Performance Category	Typical Indicators	Primary Influencing Factors	Optimization Strategies	Reference
Tensile Strength	200–365 MPa	B_4_C content, interfacial bonding, heat treatment	Hybrid reinforcement, interface modulation	[42,70]
Compressive Strength	Up to 1065 MPa	Reinforcement phase size, uniform distribution	Nano-reinforcement, severe plastic deformation	[43,61]
Hardness	Increased by 50–106%	B_4_C content, particle size	Optimized reinforcement ratio, heat treatment	[70]
Neutron Shielding	Transmission coefficient reduced by 90%	10B areal density, material thickness	High B_4_C content, gradient design	[6]
Thermal Conductivity	Increased by 46.4%	Reinforcement phase size, distribution	Large-sized particles, functional gradient	[53]
Wear Resistance	Improved by 3–20 times	B_4_C content, lubricating phase	Addition of solid lubricants	[75]
Corrosion Resistance	Decreases with increased B_4_C	Interfacial galvanic corrosion	Surface treatment, alloying	[79]

**Table 9:** Density and porosity of multi-component-reinforced Al-based composites (BN/Al, BN-CNTs/Al, B_4_C/Al).

Composite Type	Reinforcement Content	Theoretical Density (g/cm^3^)	Sintered/As-built Density (g/cm^3^)	Relative Densification (%)	Porosity (%)	Reference
BN/Al	1 wt.% BN	2.69	2.55	95.5	4.4	[36]
BN/Al	3 wt.% BN	2.68	2.60	96.8	3.1	[36]
BN-CNTs/Al	3 wt.% BN + 0.5 wt.% CNTs	2.69	2.63	97.7	2.2	[36]
B_4_C/Al (SLM)	20 wt.% B_4_C (scanning speed: 100 mm/s)	2.81	2.73	97.1	2.9	[54]
B_4_C/Al (SLM)	20 wt.% B_4_C (scanning speed: 700 mm/s)	2.81	2.39	85.0	15.0	[54]

**Table 10:** Primary Applications and Typical Cases of B_4_C/Al Composites.

Application Field	Critical Performance Requirements	Typical Application Cases	Advantages/Features	Reference
Nuclear shielding	High neutron absorption (Σa), radiation resistance	Reactor control rods, spent fuel containers	^10^B enrichment (≥19.8%), low activation	[6]
Military armor	Ballistic limit (V50), hardness (≥70 HRC)	Vehicle armor plates, personal protection	High hardness-to-density ratio (8.5 GPa·cm^3^/g)	[20]
Aerospace components	Specific strength (≥380 MPa·cm^3^/g), thermal stability	Satellite structural parts, UAV frames	Low CTE (6.5 × 10^−6^/K), vibration damping	[22,36,89]
Automotive lightweight	Wear resistance (≤3 × 10^−6^ mm^3^/Nm), cost efficiency	Brake rotors, suspension arms	40% weight reduction vs. steel	[22,90]
Thermal management	Thermal conductivity (≥180 W/m·K), dimensional stability	CPU heat sinks, power modules	Tunable CTE matching Si	[37,65,68]

## Data Availability

No new data were created or analyzed in this study. Data sharing is not applicable to this article.
